# Effect of combined clinical pathway and scenario simulation teaching on psychological stress and anxiety among tuberculosis interns: A retrospective cohort study

**DOI:** 10.1097/MD.0000000000042779

**Published:** 2025-06-27

**Authors:** Zhi Chen, Donglin Guo, Qian Shi, Zhong Zeng

**Affiliations:** a Department of Tuberculosis, Tuberculosis Medical Center, The Eighth Medical Center of PLA General Hospital, Beijing, China; b Department of Tuberculosis, Ganzhou No. 5 People’s Hospital, Jiangxi, China.

**Keywords:** clinical training, medical education, psychological stress, teaching methods, tuberculosis clinical interns

## Abstract

In the realm of medical education, clinical internship represents a pivotal phase where theoretical knowledge must be practically applied amidst real-world patient care, often leading to heightened stress and anxiety among interns. This transitional challenge is particularly acute for tuberculosis (TB) clinical interns, requiring innovative teaching methodologies. Therefore, we aim to evaluate the efficacy of integrating clinical pathway instruction with scenario-based simulation teaching in alleviating psychological stress and anxiety among TB clinical trainees. Data extraction from electronic health and educational evaluation records spanned September 2020 to September 2022, focusing on TB clinical interns. The investigation centered on 2 principal teaching methodologies used during training: the combination of clinical pathway and scenario simulation approaches. Standardized instruments quantified psychological stress and anxiety at 3 intervals – pre-teaching, post-teaching, and a 6-month follow-up. The study encompassed 70 TB clinical interns, allocating 34 to a combined teaching approach integrating clinical pathways and scenario simulations, while 36 underwent conventional teaching methodologies. Post-intervention assessments revealed significant reduction in psychological stress levels among those subjected to the combined methods (*t* = 2.522, *P* = .014). This difference was further magnified at the 6-month follow-up, with the combined teaching method group demonstrating markedly lower psychological stress levels (*t* = 3.54, *P* < .001). Also, immediately following the intervention, the combined method group experienced significantly lesser anxiety (*t* = 2.278, *P* = .026), and the beneficial effect endured through the 6-month mark (*t* = 2.41, *P* = .019). Combining clinical pathway teaching method and scenario simulation teaching method are associated with reduced psychological stress and anxiety levels among TB clinical interns.

## 1. Introduction

Medical education and training programs are essential in shaping the competencies, clinical skills, and professional development of future healthcare providers.^[1,2]^ Clinical interns, in particular, undergo a critical phase of learning and adaptation as they transition from classroom-based learning to direct patient care responsibilities.^[3]^ This transition is often accompanied by heightened levels of psychological stress and anxiety, as interns are tasked with integrating theoretical knowledge with hands-on clinical practice in real healthcare settings.^[4,5]^ The psychological well-being of clinical interns is of paramount importance, as it not only influences their individual learning experience and clinical performance but also has implications for patient care outcomes and overall healthcare delivery.^[6,7]^ Amid the evolving landscape of medical education, innovative teaching methods have emerged as potential avenues for addressing the psychological well-being of medical trainees and enhancing their educational outcomes.^[8]^ In particular, tuberculosis (TB) clinical interns face unique clinical and public health challenges in the management of TB patients, often operating in resource-limited settings with complex patient cases and diverse sociocultural factors.^[9]^ The psychological stress and anxiety associated with navigating the complexities of TB patient care, conducting sensitive discussions around diagnosis and treatment, and managing infection control measures can significantly impact the well-being and performance of TB clinical interns.^[10,11]^ As such, investigating the potential effects of innovative teaching methods, specifically the combined clinical pathway and scenario simulation teaching methods, on the psychological stress and anxiety levels of TB clinical interns is of particular relevance in addressing the unique needs of this subset of medical trainees.

The integration of clinical pathway teaching methods and scenario simulation teaching methods represents a contemporary approach to medical education that aims to provide a more immersive, experiential, and supportive learning environment for clinical interns.^[12,13]^ Clinical pathway teaching emphasizes a structured and competency-based approach to medical training, guiding learners through a predefined set of clinical experiences and skills necessary for independent practice.^[14]^ On the other hand, scenario simulation teaching involves the use of realistic clinical scenarios in a simulated environment to engage learners in active decision-making, critical thinking, and clinical skill application.^[15,16]^ By fostering a dynamic and interactive learning environment, scenario simulation teaching methods offer opportunities for repetitive practice, clinical skill refinement, and the development of clinical reasoning abilities in a controlled and supportive setting.^[9]^ The combined implementation of clinical pathway and scenario simulation teaching methods holds potential for augmenting the educational experience of clinical interns and addressing the multifaceted challenges they encounter during their training.

In this context, the present study aimed to investigate the impact of combined clinical pathway teaching methods and scenario simulation teaching methods on the psychological stress and anxiety levels of TB clinical interns. By examining the potential influence of these teaching methods on the psychological well-being of TB clinical interns, this research seeks to contribute to the broader discourse on medical education and training, with a specific focus on the psychological well-being of medical trainees in the context of managing complex infectious diseases such as TB.

## 2. Materials and methods

### 2.1. Study design and participants

The study design was involved the retrospective review of anonymized data collected from records of TB clinical interns who participated in the clinical training program between September 2020 and September 2022. The study was conducted at The Eighth Medical Center of PLA General Hospital and Ganzhou No. 5 People’s Hospital, which encompass the clinical training sites for medical education. Inclusion criteria encompass interns who consented to educational evaluations and whose data pertaining to psychological stress and anxiety assessments are available. The study did not impose any age or gender restrictions, and interns with a history of psychological disorders or those currently undergoing treatment for psychological conditions were excluded from the study to ensure the representation of a real-world clinical intern population. This study was approved by the Ethics Committee of The Eighth Medical Center of PLA General Hospital and Ganzhou No. 5 People’s Hospital in accordance with regulatory and ethical guidelines pertaining to retrospective research studies. Informed consent of patients was waived for this retrospective study due to the exclusive use of de-identified patient data, which posed no potential harm or impact on patient care.

### 2.2. Data collection

The research team conducted a comprehensive review of the electronic health records and educational evaluation records of the TB clinical interns. Relevant data pertaining to psychological stress and anxiety assessments, as well as educational evaluations, were extracted from the electronic database. To ensure the confidentiality and anonymity of the participants, all data was de-identified and coded with study-specific identification numbers. The data collection process adhered to the institutional guidelines for accessing and utilizing electronic health records for research purposes.

### 2.3. Exposure and outcome assessment

The exposure of interest in this study is the teaching method employed during the clinical training program. The 2 primary teaching methods assessed are the combined clinical pathway teaching method and scenario simulation teaching method. The choice of teaching method for each intern was determined through retrospective documentation in the training records. The primary outcome measures included psychological stress and anxiety levels assessed at specific time points, encompassing pre-teaching, post-teaching, and 6-month follow-up assessments.

This study utilized the Chinese perceived stress scale to collect data on psychological stress among intern students and evaluate the psychological stress levels of 2 groups of patients. The scale consists of 14 items, each rated on a 5-point scale ranging from “never” to “very often,” with each level corresponding to a score of 0 to 4. The total score ranges from 0 to 56, where a higher score indicates higher psychological stress. Items 4, 5, 6, 7, 9, 10, and 13 are positively worded and reverse-scored, while items 1, 2, 3, 8, 11, 12, and 14 are negatively worded and scored normally. A total score of ≤ 25 indicates lower psychological stress, while a score of ≥ 26 indicates higher psychological stress. The Cronbach alpha for this scale in the present study was 0.749, and the Kaiser–Meyer–Olkin value was 0.874. Additionally, the Hamilton anxiety scale was employed in assessing the anxiety levels of the intern students. This scale comprises 13 items, scored from 0 to 4, with a score of 7 being the threshold for anxiety. Scores ranging from 7 to 14 indicate possible anxiety, 14 to 21 represent mild anxiety, 21 to 29 signify moderate anxiety, and scores exceeding 29 indicate severe anxiety.

### 2.4. Statistical analysis

Descriptive statistics were used to summarize the baseline characteristics of the study cohort, including age, gender distribution, baseline stress and anxiety levels, and duration of the internship. The primary analysis involved the comparison of psychological stress and anxiety levels between the interns exposed to the combined teaching method and those receiving conventional teaching methods. Continuous variables were analyzed using *t*-tests or nonparametric tests, as appropriate, and categorical variables were analyzed using chi-square tests. Additionally, correlation analysis was conducted to explore the associations between the choice of teaching method and psychological stress and anxiety levels at different time points. Multivariate analysis was employed to adjust for potential confounders, such as age, gender, and duration of internship, depending on the findings of the univariate analysis. Statistical significance was defined at a 2-sided *P*-value of <.05.

## 3. Results

### 3.1. Baseline characteristics

Based on the study comparing the impact of conventional teaching method and combined teaching method on the psychological stress and anxiety of TB clinical interns, no significant differences were observed between the 2 teaching methods in terms of participants’ baseline characteristics (Table 1), including age (*t* = 1.128, *P* = .264), gender (χ^2^ = 0.201, *P* = .654), baseline stress (*t* = 0.153, *P* = .879), baseline anxiety (*t* = 0.218, *P* = .828), and duration of internship (*t* = 0.383, *P* = .703).

**Table 1:** Baseline characteristics of participants.

Parameter	Conventional teaching method (n = 34)	Combined teaching method (n = 36)	*t*/χ^2^	*P*-value
Age (yr)	22.27 ± 0.45	22.12 ± 0.63	1.128	.264
Gender (M/F)	18/ 16	22/ 14	0.201	.654
Baseline stress	35.21 ± 5.67	35.42 ± 5.81	0.153	.879
Baseline anxiety	34.89 ± 6.02	35.21 ± 6.12	0.218	.828
Duration of internship (d)	46.24 ± 1.32	46.36 ± 1.41	0.383	.703

### 3.2. Psychological stress levels

The impact of combined clinical pathway teaching method and scenario simulation teaching method on the psychological stress levels of TB clinical interns was assessed at different time points (Table 2). There were no significant differences in the pre-teaching psychological stress levels between the conventional teaching method and combined teaching method groups (*t* = 0.153, *P* = .879). However, at the post-teaching assessment, the combined teaching method group exhibited significantly lower psychological stress levels compared to the conventional teaching method group (*t* = 2.522, *P* = .014). This difference was further magnified at the 6-month follow-up, with the combined teaching method group demonstrating markedly lower psychological stress levels (*t* = 3.54, *P* < .001). These results suggest that the combined teaching method may have a substantial and lasting impact in reducing the psychological stress levels of TB clinical interns compared to the conventional teaching method.

**Table 2:** Psychological stress levels.

Time point	Conventional teaching method (n = 34)	Combined teaching method (n = 36)	*t*	*P*-value
Pre-teaching	35.21 ± 5.67	35.42 ± 5.81	0.153	.879
Post-teaching	28.76 ± 4.92	25.84 ± 4.75	2.522	.014
6 mo follow-up	24.09 ± 4.71	20.14 ± 4.63	3.54	<.001

### 3.3. Anxiety levels

The impact of combined clinical pathway teaching method and scenario simulation teaching method on the anxiety levels of TB clinical interns was investigated at different time points (Table 3). At the pre-teaching assessment, there were no significant differences in anxiety levels between the conventional teaching method group and the combined teaching method group (*t* = 0.222, *P* = .825). However, following the teaching intervention, the combined teaching method group exhibited significantly lower anxiety levels compared to the conventional teaching method group (*t* = 2.278, *P* = .026). This difference persisted at the 6-month follow-up, with the combined teaching method group showing significantly lower anxiety levels (*t* = 2.41, *P* = .019). These findings suggest that the combined teaching method is associated with a sustained reduction in anxiety levels among TB clinical interns compared to the conventional teaching method, indicating its potential benefit in addressing anxiety in this cohort.

**Table 3:** Anxiety levels.

Time point	Conventional teaching method (n = 34)	Combined teaching method (n = 36)	*t*	*P*-value
Pre-teaching	34.89 ± 6.02	35.21 ± 6.11	0.222	.825
Post-teaching	29.45 ± 5.18	26.67 ± 5.01	2.278	.026
6 mo follow-up	24.14 ± 5.36	21.09 ± 5.22	2.41	.019

### 3.4. Knowledge gain

The impact of the combined clinical pathway teaching method and scenario simulation teaching method on knowledge gain among TB clinical interns was assessed (Table 4). The pre-teaching knowledge scores did not differ significantly between the conventional teaching method group and the combined teaching method group (*t* = 0.198, *P* = .844). However, following the teaching intervention, the combined teaching method group showed a significantly higher post-teaching knowledge score compared to the conventional teaching method group (*t* = 2.451, *P* = .017). These results suggest that the combined teaching method led to a greater knowledge gain among TB clinical interns compared to the conventional teaching method, indicating its potential efficacy in improving knowledge acquisition in this cohort.

**Table 4:** Knowledge gain.

Parameter	Conventional teaching method (n = 34)	Combined teaching method (n = 36)	*t*	*P*-value
Pre-teaching score	62.51 ± 8.21	62.91 ± 8.47	0.198	.844
Post-teaching score	75.21 ± 7.85	79.89 ± 8.12	2.451	.017

### 3.5. Correlation analysis

The correlation between the different teaching methods and psychological stress levels as well as anxiety levels among TB clinical interns was examined (Table 5). The post-teaching psychological stress levels displayed a significant negative correlation with the choice of teaching method (*r* = −0.293, *R*^2^ = 0.086, *P* = .014), indicating that the combined teaching method was associated with lower post-teaching psychological stress levels. A similar negative correlation was observed at the 6-month follow-up, with the choice of teaching method significantly correlating with reduced psychological stress levels (r = −0.395, *R*^2^ = 0.156, *P* < .001). Additionally, the post-teaching anxiety levels also showed a negative correlation with the teaching method (*r* = −0.266, *R*^2^ = 0.071, *P* = .026), suggesting that the combined teaching method was associated with lower post-teaching anxiety levels. This negative correlation persisted at the 6-month follow-up for anxiety levels (*r* = −0.281, *R*^2^ = 0.079, *P* = .019). Furthermore, a positive correlation was found between the choice of teaching method and post-teaching knowledge score (*r* = 0.285, *R*^2^ = 0.081, *P* = .017), indicating that the combined teaching method was linked to higher post-teaching knowledge scores. These results suggest that the combined clinical pathway teaching method and scenario simulation teaching method may be correlated with reduced psychological stress levels, anxiety levels, and improved knowledge acquisition among TB clinical interns.

**Table 5:** correlation analysis between different teaching method and stress and anxiety level.

Parameter	*r*	*R* ^2^	*P*-value
Post-teaching psychological stress levels	−0.293	0.086	.014
6 mo follow-up psychological stress levels	−0.395	0.156	<.001
Post-teaching anxiety levels	−0.266	0.071	.026
6 mo follow-up anxiety levels	−0.281	0.079	.019
Post-teaching score	0.285	0.081	.017

## 4. Discussion

The impact of combined clinical pathway teaching method and scenario simulation teaching method on the psychological stress and anxiety of TB clinical interns has significant implications for various aspects of medical education and training.^[10,11]^ In this study, we sought to investigate the potential benefits of integrating these teaching methods in addressing the psychological well-being of clinical interns, particularly focusing on psychological stress and anxiety levels. The results of the study revealed notable associations between the combined teaching method and reduced psychological stress and anxiety levels, as well as improved knowledge acquisition among TB clinical interns. These findings indicate the potential efficacy of the combined teaching method in positively influencing the psychological well-being and educational outcomes of clinical interns.

The significant association between the combined teaching method and reduced psychological stress levels among TB clinical interns is a noteworthy finding. Medical education and training can be inherently stressful, particularly for interns who are often exposed to challenging clinical scenarios while still assimilating a vast amount of medical knowledge and skills.^[17–19]^ Consequently, interventions aimed at mitigating stress are crucial not only for the well-being of clinical interns but also for optimizing their learning and performance. The observed negative correlation between the choice of teaching method and post-teaching psychological stress levels, as well as the 6-month follow-up stress levels, suggests that the combined teaching method may offer unique advantages in managing and reducing psychological stress among clinical interns. This finding aligns with previous research that has highlighted the potential benefits of simulation-based teaching methods in reducing stress and enhancing the confidence of medical students and trainees.^[20,21]^ The immersive and interactive nature of scenario simulation teaching can provide a more controlled and supportive environment for clinical skill acquisition, potentially contributing to lower stress levels among interns compared to traditional didactic teaching methods.^[22]^ Moreover, creating a realistic but safe environment for practicing various clinical scenarios can help interns develop essential coping mechanisms and decision-making skills, consequently contributing to stress reduction and improved clinical preparedness.^[23]^ Additionally, the collaborative and experiential nature of scenario simulation teaching can foster a supportive learning environment, potentially enhancing interns’ confidence in their clinical abilities and reducing stress and anxiety associated with patient care responsibilities.^[24]^

The findings related to anxiety levels further underscore the potential benefits of the combined teaching method in addressing the psychological well-being of TB clinical interns. Anxiety is a prevalent concern in medical education, as trainees often experience high levels of pressure, uncertainty, and responsibility in their clinical roles.^[18,25]^ The observed negative correlation between the combined teaching method and post-teaching anxiety levels, as well as the 6-month follow-up anxiety levels, suggests that this teaching approach may contribute to mitigating anxiety symptoms among clinical interns. Simulation-based teaching methods, particularly those involving realistic clinical scenarios, have been recognized for their potential in reducing anxiety and enhancing clinical confidence among medical trainees.^[24,26]^ By providing opportunities for repetitive practice in a controlled setting, simulation-based methods can help interns become more familiar and comfortable with intricate clinical procedures and decision-making, potentially leading to lower anxiety levels and improved clinical performance.^[11,19]^ Additionally, the dynamic and interactive nature of scenario simulation teaching can promote active engagement and critical thinking, which are essential skills for managing anxiety-inducing situations in real clinical settings.^[10]^ The observed reduction in anxiety levels among interns exposed to the combined teaching method may reflect the beneficial impact of scenario simulation on enhancing clinical preparedness, confidence, and resilience, ultimately contributing to lower anxiety levels in both the short and long terms.

In addition to its effects on psychological well-being, the combined teaching method also demonstrated a positive association with improved knowledge acquisition among TB clinical interns. The observed higher post-teaching knowledge scores among interns exposed to the combined teaching method indicate the potential educational benefits of integrating clinical pathway and scenario simulation teaching methods. While traditional didactic teaching methods remain fundamental in disseminating medical knowledge, scenario simulation teaching offers a more engaging and experiential approach, allowing interns to apply theoretical knowledge in practical clinical contexts.^[16]^ This active participation and experiential learning can enhance knowledge retention, clinical reasoning, and problem-solving skills, ultimately leading to improved knowledge acquisition and retention among medical trainees. Moreover, the psychological safety provided by scenario simulation teaching can create an environment conducive to learning, where interns can make mistakes, receive constructive feedback, and refine their clinical competencies without the high stakes and pressure associated with real patient care.^[3,5]^ As a result, the observed higher post-teaching knowledge scores among interns exposed to the combined teaching method may reflect the educational advantages of scenario simulation teaching in enabling a more robust and lasting understanding of clinical concepts and practices.

The findings from this study contribute to the growing body of evidence supporting the beneficial effects of innovative teaching methods on the psychological well-being and educational outcomes of medical trainees.^[1,2]^ By identifying the potential benefits of a combined clinical pathway teaching method and scenario simulation teaching method, this research highlights the value of integrating diverse and interactive teaching approaches in medical education and training programs. The observed associations between the combined teaching method and reduced psychological stress and anxiety levels, as well as improved knowledge acquisition, underscore the potential of innovative teaching methods to positively impact multiple domains of medical training. The findings from this study hold the potential to inform the design and implementation of targeted educational interventions aimed at improving the psychological well-being and educational outcomes of TB clinical interns, with implications for enhancing the quality of TB patient care and the professional development of future healthcare providers. Additionally, by elucidating the potential benefits of innovative teaching methods, this research may contribute to the ongoing evolution of medical education paradigms towards a more comprehensive, supportive, and effective learning environment for medical trainees across diverse clinical specialties. Building on these findings, future research could further explore the specific mechanisms through which these teaching methods influence psychological well-being and educational outcomes.

## 5. Conclusion

In conclusion, the results of this study provide compelling evidence for the potential benefits of integrating combined clinical pathway teaching and scenario simulation teaching methods in addressing the psychological well-being and educational outcomes of TB clinical interns. The observed associations between the combined teaching method and reduced psychological stress and anxiety levels, as well as improved knowledge acquisition, shed light on the promising effects of innovative teaching approaches in medical education. By fostering a supportive and immersive learning environment, scenario simulation teaching, in conjunction with clinical pathway teaching, holds promise for not only reducing psychological distress among clinical interns but also enhancing their clinical confidence and knowledge acquisition. These findings underscore the importance of embracing diverse and interactive teaching methods in medical education and training programs to promote the well-being and competence of future healthcare professionals.

## Acknowledgments

The authors are grateful to all participants in the present study.

## Author contributions

**Conceptualization:** Zhi Chen, Zhong Zeng.

**Investigation:** Zhi Chen, Donglin Guo, Qian Shi, Zhong Zeng.

**Methodology:** Zhi Chen, Donglin Guo, Qian Shi, Zhong Zeng.

**Writing – original draft:** Zhi Chen.

**Writing – review & editing:** Zhong Zeng.
